# Cerebrovascular Response to Propofol, Fentanyl, and Midazolam in Moderate/Severe Traumatic Brain Injury: A Scoping Systematic Review of the Human and Animal Literature

**DOI:** 10.1089/neur.2020.0040

**Published:** 2020-10-13

**Authors:** Logan Froese, Joshua Dian, Carleen Batson, Alwyn Gomez, Bertram Unger, Frederick A. Zeiler

**Affiliations:** ^1^ Biomedical Engineering, Faculty of Engineering, University of Manitoba, Winnipeg, Manitoba, Canada.; ^2^ Section of Neurosurgery, Department of Surgery, Department of Medicine, Rady Faculty of Health Sciences, University of Manitoba, Winnipeg, Manitoba, Canada.; ^3^ Department of Anatomy and Cell Science, Department of Medicine, Rady Faculty of Health Sciences, University of Manitoba, Winnipeg, Manitoba, Canada.; ^4^ Section of Critical Care, Department of Medicine, Rady Faculty of Health Sciences, University of Manitoba, Winnipeg, Manitoba, Canada.; ^5^ Centre on Aging, University of Manitoba, Winnipeg, Manitoba, Canada.; ^6^ Division of Anesthesia, Department of Medicine, Addenbrooke's Hospital, University of Cambridge, Cambridge, United Kingdom.

**Keywords:** brain injury, cerebral blood flow, cerebrovascular response, fentanyl, midazolam, propofol

## Abstract

Intravenous propofol, fentanyl, and midazolam are utilized commonly in critical care for metabolic suppression and anesthesia. The impact of propofol, fentanyl, and midazolam on cerebrovasculature and cerebral blood flow (CBF) is unclear in traumatic brain injury (TBI) and may carry important implications, as care is shifting to focus on cerebrovascular reactivity monitoring/directed therapies. The aim of this study was to perform a scoping review of the literature on the cerebrovascular/CBF effects of propofol, fentanyl, and midazolam in human patients with moderate/severe TBI and animal models with TBI. A search of MEDLINE, BIOSIS, EMBASE, Global Health, SCOPUS, and the Cochrane Library from inception to May 2020 was performed. All articles were included pertaining to the administration of propofol, fentanyl, and midazolam, in which the impact on CBF/cerebral vasculature was recorded. We identified 14 studies: 8 that evaluated propofol, 5 that evaluated fentanyl, and 2 that evaluated midazolam. All studies suffered from significant limitations, including: small sample size, and heterogeneous design and measurement techniques. In general, there was no significant change seen in CBF/cerebrovascular response to administration of propofol, fentanyl, or midazolam during experiments where PCO_2_ and mean arterial pressure (MAP) were controlled. This review highlights the current knowledge gap surrounding the impact of commonly utilized sedative drugs in TBI care. This work supports the need for dedicated studies, both experimental and human-based, evaluating the impact of these drugs on CBF and cerebrovascular reactivity/response in TBI.

## Introduction

Intravenous anesthesia is used universally within care for patients with severe brain injury for its neuroprotective properties.^1^ Its use is not limited to its ability to moderate cerebral metabolism; it also provides a more stable cerebral physiology in the presence of the severe trauma.^1,2^ Despite large-scale use of intravenous anesthetic agents, the impact that these commonly employed drugs have on various aspects of cerebral physiology in critical care patients, especially those with a traumatic brain injury (TBI), is largely unknown. This is in spite of their widespread adoption and recommendation through consensus-based guidelines for the management of moderate/severe TBI.^3–5^

Of particular interest is the impact on cerebral blood flow (CBF) and cerebrovascular reactivity of such sedative agents in TBI care, as current clinical guidelines focus on improving cerebral perfusion, CBF, and end-organ nutrient delivery.^3,6–9^ The body of literature surrounding the link between impaired cerebrovascular reactivity and poor patient outcome after TBI is growing,^10–14^ with data suggesting that in modern TBI care much of the ongoing cerebral physiological insult seen is dominated by impaired cerebrovascular reactivity.^9,12,13,15^ Further, cerebrovascular reactivity-based individual cerebral physiological targets, such as optimal cerebral perfusion pressure (CPPopt)^8,16–18^ or individual intracranial pressure (iICP) thresholds,^19,20^ are emerging as novel methods to personalize treatment in TBI. Understanding the effects these commonly employed sedative agents have on CBF/cerebrovascular reactivity in the patient with severe TBI is a pivotal step in advancing personalized care.

The goal of this study was to perform a systematically conducted scoping review of all available literature on the impact of three commonly employed sedative agents used in moderate/severe TBI care (i.e., propofol, fentanyl, and midazolam) on cerebrovascular responsiveness/CBF response in human patients with moderate/severe TBI and animal TBI models.

## Methods

A systematic review of the available literature was conducted using the methodology outlined in the *Cochrane Handbook for Systematic Reviews of Interventions*.^21^ The data were reported in line with the Preferred Reporting Items for Systematic Reviews and Meta-Analyses (PRISMA).^22^ Supplementary Table S1 provides the PRISMA checklist. The review questions and search strategy were decided upon by the supervisor (F.A.Z.) and primary author (L.F.).

### Ethical considerations

All articles are from previously published journals and have been vetted by their respective journals.

### Search question, population, and inclusion and exclusion criteria

The question posed for systematic review was: “What is the effect of exogenous systemically administered propofol, fentanyl, or midazolam on the cerebrovascular response/CBF in human patients with moderate/severe TBI and animal models with TBI?” All studies, prospective and retrospective, of any size, based on humans and animals were included.

The primary outcome measure was the impact on CBF or the cerebrovascular responsiveness as documented by any objective means of CBF/cerebrovascular reactivity assessment, including continuous measures and neuroimaging-based or blood sampling-based techniques.

All original studies, whether prospective or retrospective, of all sizes, of any human age category or animal TBI model design, with the use of propofol/fentanyl/midazolam, and with formal documentation of cerebrovascular response/CBF during administration were eligible for inclusion in this review. Exclusion criteria were as follows: mild TBI literature, non-TBI human literature, being a non-English language study, or conducting CBF mediation with a substance other than propofol/fentanyl/midazolam.

### Search strategy

MEDLINE, BIOSIS, EMBASE, Global Health, SCOPUS, and the Cochrane Library from inception to May 2020 were searched using individualized search strategies for each database. The search strategy for MEDLINE can be found in Supplementary Table S2, and a similar search strategy was used for the other databases. Finally, the reference lists of reviewed articles on the cerebral blood vessels/CBF response to propofol, fentanyl, and midazolam were examined to ensure no references were left out.

### Study selection

Using two reviewers (L.F. and J.D.), a two-step review of all articles returned by our search strategies was performed. First, the reviewers independently screened all titles and abstracts of the returned articles to decide whether they met the inclusion criteria. Second, full text of the chosen articles was assessed to confirm whether the articles met the inclusion criteria and that the primary outcome of CBF/cerebrovascular response to propofol, fentanyl, and midazolam was documented. Any discrepancies between the two reviewers were resolved by a third party (F.A.Z.).

### Data collection

Data were extracted from the selected articles and stored in multiple electronic databases to ensure data integrity.

### Human studies

Data fields included the following: number of patients/animals, type of study, patient/model characteristics, the goal of the study, dose of anesthetic administered, type of anesthetic administered, technique of CBF/vasculature assessment, CBF/cerebral vasculature response to drug, other outcomes, and general conclusions.

### Bias assessment

Given the goal of this review was to provide a comprehensive scoping overview of the available literature, a formal bias assessment was not conducted.

### Statistical analysis

A meta-analysis was not performed in this study because of the heterogeneity of model types, study designs, and data.

## Results

### Search results and study characteristics

**FIG. 1.**
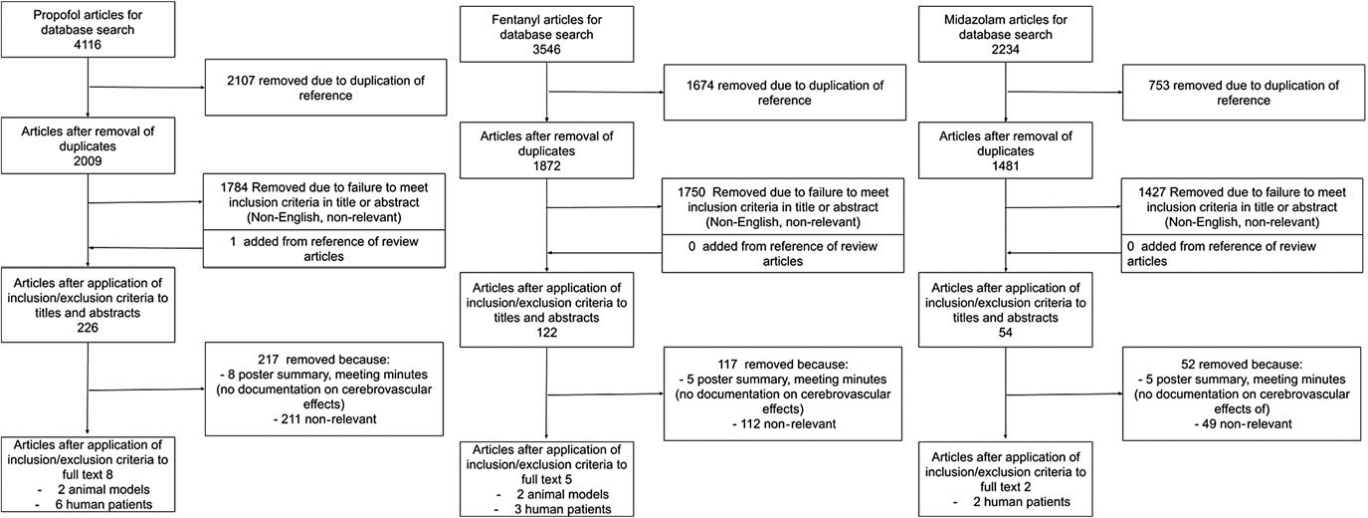
PRISMA flow diagram. PRISMA, preferred reporting in systematic reviews and meta-analysis.

The results of the search strategy across all databases and reference sections of articles are summarized in Figure 1. Overall, a total of 9896 articles were identified, all from the databases searched. A total of 4534 were removed because of duplication of references, leaving 5362 to review. By applying the inclusion/exclusion criteria to the title and abstract of these articles, we identified 400 articles that fit these criteria. One article was added from reference sections of pertinent review articles, leaving a total of 401 articles to review. The portable document formats (PDFs) of these 401 were then gathered. Applying the inclusion/exclusion criteria to these PDFs, only 14 articles were found eligible for inclusion in the systematic review.

Within the 14 TBI studies identified, there were 10 human TBI studies, and 4 animal TBI model studies. In the 10 human TBI studies, all the patients suffered a moderate/severe TBI, with human patients having a Glasgow Coma Scale (GCS) score of 12 or less on presentation. All studies measured CBF response to propofol, fentanyl, midazolam, and other agents: 5 used arterio-jugular differences of oxygen (AVDO_2_),^10,23–26^ 2 studies used a Xenon^133^ diffusion technique,^27,28^ 1 study used laser speckle imaging,^29^ 1 study used radiolabeled microsphreres,^30^ 4 studies used transcranial-Doppler flow velocity,^10,26,28,31^ and 4 studies used CPP/PO_2_^24,32–34^ as a surrogate for CBF.^35^ There were 3 studies that evaluated cerebrovascular reactivity/responsiveness, as measured by response of CBF to CO_2_ reactivity^25,26^ or a variety of other methods that used CBF and CBF velocity (CBFv).^28^ Regarding specific sedative agent studies, there were 8 studies that used propofol (2 of which used rat models^29,31^), 5 studies that used fentanyl (1 used rats^36^ and 1 used cats^30^), and 2 studies that used midazolam. The characteristics of the studies can be found in Table 1, Table 2, and Supplementary Table S3.

**Table 1.** Included Studies: General Characteristics and Study Goals

References	No. patients/animals	Study type	Article location	Mean age	Patient/Animal characteristics	Primary and secondary goal of study
*Human studies*						
Lee et al.^28^	28 patients	Prospective cohort study	Journal	33 ± 13 years	TBI patients with GCS <7	Primary: Assess influence of CO_2_ reactivity, pressure autoregulation, and metabolic suppression reactivity after head injury
						Secondary: Compare hemisphere response in CBF velocity
Steiner et al.^10^	10 patients	Prospective cohort study	Journal	35 ± 12 years	TBI patients with GCS score ≤12, 7 men and 3 women, with evacuated mass lesion in 8, diffuse injury 2 in 2, large bilateral lesions in 5, and 7 had a craniectomy	Primary: Effect of propofol plasma concentration on pressure autoregulation
James et al.^34^	8 patients	Prospective randomized unblinded single crossover observational pilot study	Journal	Not mentioned	4 patients with TBI, 3 with subarachnoid hemorrhage and one with intracerebral hemorrhage with median GCS score of 6.1 and acute physiology and chronic health evaluation 2 scores of 13.5	Primary: Effects of dexmedetomidine and propofol on cerebral physiology in acute brain injury patients
Johnston et al.^23^	10 patients	Prospective cohort study	Journal	21–53 years	TBI patients with GCS score 3–9, 8 patients with evacuated mass lesion and 2 with diffuse injury 2	Primary: Assess the effect of propofol on cerebral oxygenation and metabolism in head-injured patients
						Secondary: Use propofol to achieve EEG burst suppression and evaluate overall effects on ischaemic burden
Pinaud et al.^27^	10 patients	Prospective cohort study	Journal	14–40 years	TBI patients with GCS score ≤6	Primary: Effects of propofol on cerebral hemodynamics and metabolism in TBI patients
Tanguy et al.^33^	30 patients	Retrospective cohort study	Journal	35 ± 18 years	TBI patients with acute physiological score 2–4 with mean GCS score 5	Primary: Compare the cerebral microdialysis effects of propofol vs midazolam in TBI patients
Albanèse et al.^24^	6 patients	Randomized unblended crossover study	Journal	20–44 years	TBI male patients with GCS score 4–8	Primary: Assess the effects of sufentanyl, fentanyl, and alfentanil on cerebral hemodynamics
de Nadal et al.^26^	30 patients	Randomized crossover study	Journal	30 ± 13 years	TBI patients with GCS score ≤8	Primary: Evaluate the cerebral hemodynamic effects of morphine and fentanyl in TBI patients
						Secondary: Correlation of morphine and fentanyl to cerebral autoregulation
de Nadal et al.^25^	30 patients	Prospective cohort study	Journal	30.2 ± 13.2 years	TBI patients with GCS score ≤8	Primary: Evaluate the effects of fentanyl in TBI patients
Papazian et al.^32^	12 patients	Prospective cohort study	Journal	14–44 years	TBI patient with GCS score ≤6	Primary: Effect of midazolam on ICP and CPP in TBI
						Secondary: Evaluate cerebral damage by CPP increase
*Animal studies*						
Feuerstein et al.^29^	28 rats	Four-arm study	Journal	Not applicable	Male Wistar rats initially anesthetized with isoflurane, TBI method not mentioned	Primary: Evaluation of different methods to detect CBF and tissue deterioration after TBI
Kahveci et al.^31^	16 rats	Two-arm study	Journal	Not applicable	Female Wistar rates with hypothermia and TBI caused from accelerated impact	Primary: Effects of propofol and isoflurane on cerebral hemodynamics during hypothermic conditions
Bedell et al.^30^	17 cats	Two arm-study	Journal	Not applicable	Cats initially anesthetized with isoflurane and nitrous oxide, then TBI was induced with a fluid percussion injury	Primary: Influence of fentanyl on CBF during hypotension after TBI
Statler et al.^36^	51 rats	Two-arm study	Journal	Not applicable	Sprague-Dawley rats initially anesthetized with nitrous oxide and isoflurane, TBI was induced with control cortical impact	Primary: Evaluate the effects of isoflurane and fentanyl in TBI rats
						Secondary: Lesion volumes after TBI in rats

CBF, cerebral blood flow; CPP, cerebral perfusion pressure; EEG, electroencephalogram; GCS, Glasgow Coma Scale; TBI, traumatic brain injury.

**Table 2.** Sedation Treatment and Cerebrovascular Response: Summary of Study Details

References	medication and dose	CBF/Cerebrovascular response	Limitations	Conclusions
*Human studies*				
Lee et al.^28^	Propofol: 1 mg/kg	Metabolic reactivity was induced through propofol burst suppression -CPP increase by 5% (*p* < 0.01) -SjvO_2_ increase by 3% (*p* < 0.01) -MAP was constant -MCAv in most models decrease by 30%, CBF also demonstrated a decrease but was not significant -Trend to deteriorate vasoreactivity with 20% of patients having reduced response PCO_2_ and PO_2_ levels were controlled through ventilation	MAP during burst suppression was maintained with phenylephrine, which may interfere with CBF	Propofol through metabolic suppression decreased CBFv
Steiner et al.^10^	Propofol: 3-4 mg/kg/h	Propofol -Higher doses decreased MCAv by 8% -Little change to CPP or AVDO_2_ -MAP remained relatively constant -Static rate of autoregulation on average decreased from 56 ± 36 to 28 ± 35% but increased in some patients PCO_2_ and PO_2_ levels were controlled through ventilation	MAP was maintained with norepinephrine, which may interfere with CBF	Propofol decreases MCAv, which is a surrogate measure of CBFv
James et al.^34^	Propofol: 25.5 μg/kg/min Dexmedetomine: 0.54 μg/kg/h	Propofol -Slight decrease in ICP and no change in PbtO_2_ although both lacked statistical significance -CPP increased during and fell after injection by about 6% -Lactate/Pyruvate ratio increased drastically after injection -CBF had minimal changes, based on limited response in ICP and PO_2_ Dexmedetomine -A slight increase in ICP with no change in PbtO_2_ though both lacked statistical significance -CPP fell slightly by 2% -Lactate/Pyruvate ratio increased drastically after injection -CBF had minimal changes, based on limited response in ICP and PO_2_ PCO_2_ and PO_2_ levels were controlled through ventilation From ICP and CPP, MAP can be assumed to be near constant	Based on limited number of patients	Propofol demonstrated little to no effect on CBF derived from the ICP/PO_2_ comparison
Johnston et al.^23^	Propofol: 3-4 mg/kg/h	Propofol -AVDO_2_, ICP, and PCO_2_ all slightly decreased as compared with baseline values -PO_2_ and PbtO_2_ slightly increased -CPP and lactate/pyruvate ratio had little variation -All changes were not significant	ICP, CPP, and MAP can be assumed to be near constant	Propofol demonstrates a slight non-significant increase to CBF in the setting of TBI
Pinaud et al.^27^	Propofol: 2 mg/kg then 150 μg/kg/min (3-5 μg/mL)	Propofol -rCBF decrease by 25% (*p* < 0.01) -ICP decreased by 18% (*p* < 0.001), this decrease was then inverted after propofol infusion ceased -CPP dropped by28% (*p* < 0.001) -AVDO_2_ decreased by (6%) but was not significant -CVR increased then decrease as a result from propofol although this was not significant apart from 1 patient -PCO_2_ remained constant at 33 ± 2 mm Hg	Large variation within individual patients was not accounted for	Propofol caused a varying decrease in rCBF in all patients; this was associated with a decrease in ICP and CPP This indicates that rCBF drop is caused by MAP decrease
Tanguy et al.^33^	Propofol: 1 mg/kg/h and increased by same increment with 5 mg/kg/h being max Midazolam: 0.03 mg/kg/h and increased by 0.01 mg/kg/h	Propofol -ICP of 19 ± 12 mm Hg -PO_2_ of 97 ± 2% -PCO_2_ of 38 ± 7 mm Hg -CPP of 73 ± 11 mm Hg -MAP of 91 ± 11 mm Hg -CBF had minimal changes, based on limited response in CPP and PO_2_ Midazolam **-**ICP of 20 ± 12 mm Hg -PO_2_ of 98 ± 1% -PCO_2_ of 35 ± 10 mm Hg -CPP of 73 ± 11 mm Hg -MAP of 100 ± 16 mm Hg -CBF had minimal changes, based on limited response in CPP and PO_2_ PCO_2_ and PO_2_ levels were controlled through ventilation No difference was seen in the lactate/pyruvate ratio was seen	MAP was maintained with catecholamines, which may interfere with CBF Therapeutic goals and sedation levels were independent from microdialysis biomarkers	Using the CPP/PO_2_ to find CBF, it is indicated that propofol and midazolam are near identical in CBF effect, both demonstrating no significant response
Albanèse et al.^24^	Sufentanil: 1 μg/kg then 0.005 μg/kg/min Alfentanil: 100 μg/kg then 0.7 μg/kg/min Fentanyl: 10 μg/kg then 0.075 μg/kg/min	Sufentanil, alfentanil, and fentanyl -Initial increase ICP (25%) then after 60 min ICP returned to baseline -CPP decreased by 41% (*p* < 0.05) -SvjO_2_ remained relatively unchanged -Based on CPP/ SvjO_2,_ CBF was indicated to increase -Based on CMRO_2_/AVDO_2,_ CBF slightly decreased PCO_2_ levels were maintained between 32 and 35 torr and CPP stayed between 27 to 37 mm Hg PCO_2_ and PO_2_ levels were controlled through ventilation No changes in lactate-oxygen index or MAP	Based on limited number of patients	Sufentanil, alfentanil, and fentanyl had a slight increase and decrease to CBF found through the surrogate measure of CPP/SvjO_2_ and CMRO_2_/AVDO_2_ although it was not maintained or significant
de Nadal et al.^26^	Morphine: 0.2 mg/kg Fentanyl: 2 μg/kg	Morphine -Slight increase in CBF (10%) with no change in MCAv -When comparing autoregulation there was little difference in MCAv; however for CBF, impaired autoregulation demonstrated lower overall response (7%) then intact autoregulation (13%) Fentanyl -Slight increase in CBF (10%) and a slight decrease in CBFv (10%) -There was little difference in impaired vs. intact autoregulation in CBF and CBFv response All changing in AVDO_2_ were adjusted for PCO_2_ levels MAP remained relatively constant in all groups Slight decrease in CPP associated with ICP then increase to baseline	CBF was approximated or found from the MCA MAP was maintained with phenylephrine, which may interfere with CBF	Fentanyl showed little change in CBF in any group whether with intact or impaired autoregulation although the direct measurement by CBFv and 1/AVDO_2_ contradicted in response Morphine showed a slight increase in CBF with no significant change in CBFv. Intact autoregulation had a higher response then impaired autoregulation in any group whether intact or impaired autoregulation although the direct measurement by CBFv and 1/AVDO_2_ contradicted in response
de Nadal et al.^25^	Fentanyl: 2 μg/kg	Fentanyl -ICP: Increased then slowly decreased in both the group with intact and impaired autoregulation -CPP: Moderately decreased by 6% -AVDO_2_ initially decreased (11%) then returned to the baseline at 60 min but was not significant MAP showed a similar decrease as CPP All changing in AVDO_2_ were adjusted for PCO_2_ levels		Fentanyl showed a decrease in CPP with a small increase in 1/AVDO_2_ as a surrogate measure for CBF; this was a small and nonsignificant increase Fentanyl had little influence on autoregulation based on limited differences
Papazian et al.^32^	Midazolam: 0.15 mg/kg	Midazolam -Reduced CPP by 26% (*p* < 0.0001) -Non-significant change to ICP >18 mm Hg before TBI, when ICP <18 mm Hg before TBI an increase in ICP was observed (20%) -CBF had little change apart from mentioned ICP <18 mm Hg in which case midazolam caused a slight increase in CBF (10%) PCO_2_ and PO_2_ were measured and maintained	CBF assumed through CMRO_2_ coupling	CBF had little change apart from the mentioned ICP <18 mm Hg, in which case midazolam caused a slight increase in CBF Midazolam was assumed to have limited influence on autoregulation due to limited difference in ICP groups
*Animal studies*				
Feuerstein et al.^29^	Isoflurane at 2% Propofol: 33 to 53 mg/kg/h	Isoflurane -rCBF increased initially with injection then returned to baseline after 1 min (19.8 ± 27.2%) Propofol -rCBF increased initially with injection then returned to baseline after 1 min (27.5 ± 38.2%) -Atrial diameter decrease of 50% where isoflurane had no response Blood gasses were maintained through ventilation MAP had little change in each group	Limited number of subjects	There was little response in rCBF in both groups, with propofol demonstrating a constriction of cerebral pial vessels
Kahveci et al.^31^	Propofol: 12 mg/kg/h Isoflurane: 0.9 ± 0.04%	Propofol -Decrease ICP from 50% (*p* < 0.01) -CPP increased by 10% -No significant change to PO_2_, CBFv, or MAP Isoflurane -No significant effect on CBFv, ICP, or PO_2_ -MAP and CPP decrease over time by 30% Blood gasses were maintained through ventilation	Subjects were also in a hypothermic state, which influences CBF	Despite the limited result, it was indicated that propofol is the better choice in hypothermic conditions, with no response in CBFv The limited CBF effects of isoflurane are exaggerated by hypothermia indicating that isoflurane either caused no change or an increase in CBF
Bedell et al.^30^	Isoflurane: 1-1.5% Fentanyl: 50 μg/kg/h	Isoflurane -ICP increased -CPP decreased by 7% then returned to baseline -MAP, CBF, and CVR remain relatively constant Fentanyl -ICP decreased then slightly increased -CPP decrease by 30% -MAP decreased from 30% -CBF decreased by 22% at 75 min then increased to baseline -CVR decreased by 28% EEG, ICP, PCO_2_, PO_2_, pH, and temperature were similar between groups	Surgery may influence CBF	In the presence of hypotension fentanyl demonstrated a prevention of CBF indicating the fentanyl may increase CBF; along with this there was a decrease in CVR indicating an vasoconstrictive effect Isoflurane had little influence on cerebral vasculature
Statler et al.^36^	Isoflurane at 4% then reduced to 1% Fentanyl: 50 μg/mL then 50 μg/kg/h	Fentanyl MAP was higher than isoflurane; however during infusion the MAP and CPP remained constant throughout the experiment CPP after 4 h was greater in fentanyl than isoflurane group by10%, but both constant CBF was 2 to 3 times higher in isoflurane then fentanyl group PO_2_ and PCO_2_were controlled by ventilation	Subjects also sedated with nitrous oxide	The increase in CPP by isoflurane indicates that CBF is increased in contrast to the fentanyl demonstrating only minor change in CPP and therefor demonstrated little effect to CBF

AVDO_2_, arterio-jugular venous oxygen differences; CBF, cerebral blood flow; CBFv, cerebral blood flow velocity; CMRO_2_, cerebral metabolic rate of oxygen; CPP, cerebral perfusion pressure; CVR, cerebrovascular resistance; EEG, electroencephalogram; h, hour; ICP, intracranial pressure; MAP, mean arterial pressure; MCA, middle cerebral artery; MCAv, middle cerebral artery velocity; min, minute; mm Hg, millimeters of mercury; PbtO_2_, brain tissue oxygen tension; PCO_2_, partial pressure of carbon dioxide; PO_2_, partial pressure of oxygen; rCBF, regional cerebral blood flow; sec, second; SvjO_2_, jugular venous oxygen saturation; TBI, traumatic brain injury.

### Propofol, fentanyl, and midazolam impact on objectively measured CBF

The following subsections provide a narrative summary of the impact of propofol, fentanyl, and midazolam administration on objectively measured cerebrovascular response/CBF in human patients followed by a brief summary of the four animal model studies. A summary of main study results can be found in Table 2, with more details for the interested reader in Supplementary Table S3. Of note, the following sections describe the trends presented in the parent articles. In all the human studies but one,^34^ partial pressure of carbon dioxide (PCO_2_) levels were either maintained or accounted for in cerebral response. PO_2_ was controlled in all studies through constant ventilation parameters. MAP was maintained at a constant level for most of these human studies, except for three studies where MAP was changed due to the sedative agent.^25,27,32^

#### Propofol

Within the six studies^10,23,27,28,33,34^ that evaluated propofol and CBF in human patients with TBI, most had a non-significant change in CBF. However, one study had a trend toward decrease to regional CBF when measured through a Xenon^133^ diffusion technique. Although it should be noted that there was also a significant drop in CPP and MAP, which could account for the decrease in CBF seen.^27^ Also, in this study individual patient responses were measured, demonstrating that most patients had a drop in CBF by at least 10 mL/100 g/min^27^; further, in one patient cerebrovascular resistance (CVR; measured by CPP/CBF) was found to increase by 90% from baseline values (other patients had a limited response).

Two other studies displayed a non-significant response in CBF to propofol. Using transcranial-Doppler (TCD) to measure middle cerebral artery velocity (MCAv; which is a surrogate measure of CBF), these studies found the MCAv trended toward a decrease during propofol administration.^10,28^ In contrast to this CBFv change, CBF measured through AVDO_2_ methods demonstrated little response to propofol infusions.^10^ MAP, PCO_2_, and PO_2_ were relatively constant throughout, in both studies.

Finally, the three remaining studies demonstrated a non-significant CBF response to intravenous propofol administration. However, they did demonstrate a trend toward a decrease in CPP with no change in PO_2_. Such CPP and PO_2_ responses may indicate a decrease in CBF, based on CPP/PO_2_ as a surrogate measure for CBF.^23,33,34^ CPP and MAP remained unchanged in these studies.

#### Fentanyl

Within the three studies^24–26^ that evaluated the CBF effects of fentanyl in patients with TBI, all three had a non-significant response to fentanyl. However, there was a trend toward a decrease in CBFv found through TCD,^26^ with this drop found to be similar in patients with intact and impaired autoregulation (autoregulation was measured by comparing response of CBF with CO_2_ reactivity^37^). In contrast to the CBFv decrease seen in these studies, a trend toward a CBF increase was demonstrated with an increase in 1/AVDO_2_ (surrogate measure for CBF). This difference in CBF and CBFv response remained similar in patients with intact and impaired cerebral autoregulation.^25,26^ The PCO_2_ in these studies was between 29 and 35 torr, and MAP remained relatively unchanged during CBFv measurements.

#### Midazolam

In the two studies that evaluated CBF and midazolam in patients with TBI, there was a non-significant response to midazolam in CPP, PO_2_, and CBF. Although in one study the CPP and PO_2_ values were slightly higher in the midazolam group, compared with the propofol group.^33^ The second study demonstrated that midazolam decreases MAP by over 15 mm Hg, with patients who had an ICP <18 mm Hg before infusion demonstrating a slight increase in CPP.^32^ No definitive conclusions regarding the CBF/cerebrovascular reactivity response of midazolam can be made at this time.

### Animal studies

In the four animal studies two compared propofol with isoflurane^29,31^ and the other two compared fentanyl with isoflurane.^30,36^ In all studies PO_2_ and PCO_2_ remained constant in all models. In the two studies in which propofol was evaluated in rat models, MAP was relatively constant. Both studies demonstrated a decrease in CBFv (measured through TCD of the MCA)^31^ or a decrease in CBF measured through laser speckle imaging with propofol administration.^29^ One of these studies had ICP drastically decreasing from 18 ± 2 to 7 ± 1 mm Hg (CPP decrease of 10%),^31^ and the other demonstrated a constriction of pial cerebral vessels by 50% (through direct visualization of vessels).^29^

In the two remaining animal studies, the effects of fentanyl on CBF was evaluated. Both studies found the fentanyl groups displayed lower CBF and CPP values compared with the isoflurane groups, although CPP did trend toward increasing with fentanyl administration.^30,36^ The one study with feline models found fentanyl decreased MAP from 120 to 80 mm Hg with a significant drop in CBF (measured through radiolabel microspheres) and a slight decrease in CVR (calculated from MAP/CBF).^30^ Whereas the other study with rodents found that the fentanyl group had a CBF value that was 2 to 3 times lower than that in the isoflurane group, although the technique used and true value of CBF were not indicated.^36^

## Discussion

Through this systematically conducted scoping review of the literature surrounding the impact of propofol, fentanyl, and midazolam on CBF/cerebrovascular response in human and animal TBI, we have identified a significant knowledge gap. Although 14 studies were identified, they all suffered from some significant limitations, which restricted our ability to derive concrete conclusions regarding the CBF/cerebrovascular effects of these sedative agents. However, some general trends were seen in these studies.

First, in the studies identified propofol had a tendency to decrease CBF^23,27,34^ and CBFv.^10,28^ This has been previously described in healthy patients.^2,38^ However, it must be acknowledged that with the reduction in CPP seen in some of these studies with propofol, this alone may account for the CBF reductions.^27^ Further, some of the propofol studies estimated CBF using the 1/AVDO_2_ method, which is predicated on a relatively constant cerebral metabolic rate of oxygen (CMRO_2_).^10,23^ This may be the case in healthy patient populations, but likely does not hold true in the setting of TBI, where both regional and global changes in CMRO_2_ may fluctuate. Further, literature exists suggesting propofol may alter flow-metabolism coupling,^39^ further muddying the interpretation of CBF using the 1/AVDO_2_ technique. As such, no conclusive comments regarding the impact of propofol in CBF can be made at this time in patients with TBI, highlighting the need for future work.

Second, a decrease in CPP after fentanyl was seen in these TBI studies^24,25^; this has been commented on in past review articles.^2,38^ Along with this, there was a limited response in CBF in the three TBI studies with PCO_2_ being constant through the studies, indicating that fentanyl has little influence on CBF in the setting of ventilatory and cardiovascular support/control seen during treatment in an intensive care unit (ICU). In the animal models there was a decrease in CBF seen with fentanyl administration, compared with isoflurane, although the true influence of response is hard to identify. In one of the animal studies the decrease in CBF occurred with a concurrent decrease in MAP.^30^ Thus, as with propofol, we are limited in the conclusions that can be made, although there appears to be a no significant impact on CBF.

Third, midazolam was only evaluated in two studies with patients with TBI where CBF was objectively assessed and did not appear to have any significant impact on CBF. In one study there was a significant decrease in MAP from 89 to 71 mm Hg with a non-significant response to CBF.^32^ In healthy patients, midazolam has been documented to decrease CBF and increase in CPP.^2^ Based on the studies identified, it appears that in the setting of cardiorespiratory control in the ICU, midazolam does not appear to significantly impact CBF, although it must be acknowledged that further work is required in this area.

Finally, there was a limited response in CVR to administration of sedative agents. For example, propofol was found to have limited effects on CVR (CPP/CBF), with one patient having a significant response in CVR.^27^ Similarly, there was in one animal study that analyzed cerebral pial vessel response to propofol through direct visualization; vessels constricted by 50% as compared with baseline diameter.^29^ Whereas, fentanyl found a trend toward a decrease in CVR in one animal study, from 1.68 ± 0.46 to 1.21 ± 0.58 (as estimated through CPP/CBF).^30^

### Limitations

As mentioned above, the identified literature carries significant limitations, which hinder our ability to make conclusive statements regarding the CBF/cerebrovascular response of propofol, fentanyl, and midazolam in moderate/severe TBI. First, the literature body is low in number, consisting mainly of small case series with limited sample sizes. As well, many studies only demonstrated a weak non-significant response, which could be influenced by publication bias, therefore only trends may be commented on. Second, the studies were heterogeneous in nature, with different dosing and co-administration of medications. Further, some patients were on vasopressor drugs to support MAP and CPP during the recorded CBF response. These drugs have known cerebral vasoconstrictive properties and may therefore have confounded the results. Third, most studies employed the 1/AVDO_2_ method for CBF estimation. This method estimated CBF under the assumption of relatively fixed CMRO_2_. This may be the case in non-TBI patient populations but does not hold true in the setting of moderate/severe TBI. Similarly, propofol is known to impact flow-metabolism coupling in the brain^40,41^ and systemic blood pressure changes could have caused the CBF response in many of these studies.

These outlined limitations of the CBF measurement technique further limit our ability to interpret if these agents have a true impact on CBF. As well, CBFv methods to evaluate MCAv make the assumption that medium/large vessel changes in CBFv reflect downstream CBF/cerebrovascular responses. Finally, there is a lack of recorded high temporal physiology responses of each drug with respect to CBF, relying mainly on serological information for CBF estimation. Thus, the true temporal CBF/cerebrovascular response to these sedative agents in moderate/severe TBI remains unknown.

### Future directions

It is clear from this review that knowledge of the impact of commonly administered sedative agents on CBF/cerebrovascular response in TBI is limited. As such, we believe this review both highlights the knowledge gap and provides evidence to support further work in this area. Future investigations would benefit from both experimental animal TBI models and *in vivo* human studies in TBI. Both types of research require the use of continuous high temporal frequency CBF/cerebrovascular reactivity measurement techniques. These data would need to be time-linked to medication dosing information, to provide the optimal platform for exploring the temporal impact of such sedation agents on CBF/cerebrovascular reactivity. A multi-modal cerebral physiological monitoring approach would be preferred, employing ICP, brain tissue oxygen tension (PbtO_2_), thermal diffusion CBF, near-infrared oximetry, and cerebral microdialysis. Similarly, objective assessments of sedation depth, such as via processed electroencephalogram (EEG) data, may remove the uncertainty around individual dose-response to sedative agents. Capturing, curating, and analyzing such data require a multi-disciplinary team, consisting of clinicians, biomedical engineers, physiologists, and data scientists, like those formed by research networks such as CENTER-TBI in Europe^42,43^ and CAHR-TBI in Canada.^44^ Leveraging advances in machine learning may facilitate analysis of complex data that would be captured in these large collaborative networks.

## Conclusion

There were a limited number of articles objectively documenting the CBF/cerebrovascular response of propofol, fentanyl, and midazolam in human patients with moderate/severe TBI and in animal TBI models. All studies suffered from significant limitations and small sample sizes, limiting the conclusions that can be drawn. In general, none of the agents had a significant impact on estimated CBF in the TBI populations described. This review highlights a significant knowledge gap present regarding the CBF/cerebrovascular response of these sedative agents in moderate/severe TBI, emphasizing the need for future dedicated experimental and human studies.

## Supplementary Material

Supplemental data

Supplemental data

Supplemental data

## Abbreviations Used

AVDO_2_: arterio-jugular differences of oxygen
CBF: cerebral blood flow
CBFv: CBF velocity
CMRO_2_: cerebral metabolic rate of oxygen
CPPopt: optimal cerebral perfusion pressure
CVR: cerebrovascular resistance
EEG: electroencephalogram
GCS: Glasgow Coma Scale
ICU: intensive care unit
iICP: individual intracranial pressure
MAP: mean arterial pressure
MCA: middle cerebral artery
MCAv: middle cerebral artery velocity
PbtO_2_: brain tissue oxygen tension
PCO_2_: partial pressure of carbon dioxide
PDF: portable document format
rCBF: regional cerebral blood flow
SvjO_2_: jugular venous oxygen saturation
TBI: traumatic brain injury
TCD: transcranial-Doppler
