# Supplementary material for: Cerebrovascular Response to Propofol, Fentanyl, and Midazolam in Moderate/Severe Traumatic Brain Injury: A Scoping Systematic Review of the Human and Animal Literature
Source: Neurotrauma Rep. 2020 Oct 13;1(1):100–12. doi: 10.1089/neur.2020.0040 (PMC7685293; doi:10.1089/neur.2020.0040)
Supplement: Supplemental data [file Supp_Table2.docx]

Appendix B. Ovid search

| 1 | Cerebrovascular Circulation.mp. [mp=ab, ti, ot, bt, hw, id, cc, nm, fx, kf, ox, px, rx, ui, sy] |
| --- | --- |
| 2 | Cerebral Blood Flow.mp. [mp=ab, ti, ot, bt, hw, id, cc, nm, fx, kf, ox, px, rx, ui, sy] |
| 3 | Circulation.mp. [mp=ab, ti, ot, bt, hw, id, cc, nm, fx, kf, ox, px, rx, ui, sy] |
| 4 | Cerebral Perfusion Pressure.mp. [mp=ab, ti, ot, bt, hw, id, cc, nm, fx, kf, ox, px, rx, ui, sy] |
| 5 | Circulation, Cerebrovascular.mp. [mp=ab, ti, ot, bt, hw, id, cc, nm, fx, kf, ox, px, rx, ui, sy] |
| 6 | CBF.mp. [mp=ab, ti, ot, bt, hw, id, cc, nm, fx, kf, ox, px, rx, ui, sy] |
| 7 | [cbfv.mp](http://cbfv.mp/). [mp=ab, ti, ot, bt, hw, id, cc, nm, fx, kf, ox, px, rx, ui, sy] |
| 8 | [cpp.mp](http://cpp.mp/). [mp=ab, ti, ot, bt, hw, id, cc, nm, fx, kf, ox, px, rx, ui, sy] |
| 9 | Cerebral Homeostasis.mp. [mp=ab, ti, ot, bt, hw, id, cc, nm, fx, kf, ox, px, rx, ui, sy] |
| 10 | cerebral auto [regulation.mp](http://regulation.mp/). [mp=ab, ti, ot, bt, hw, id, cc, nm, fx, kf, ox, px, rx, ui, sy] |
| 11 | Intracranial Pressure.mp. [mp=ab, ti, ot, bt, hw, id, cc, nm, fx, kf, ox, px, rx, ui, sy] |
| 12 | Intracerebral Pressure.mp. [mp=ab, ti, ot, bt, hw, id, cc, nm, fx, kf, ox, px, rx, ui, sy] |
| 13 | Subarachnoid Pressure.mp. [mp=ab, ti, ot, bt, hw, id, cc, nm, fx, kf, ox, px, rx, ui, sy] |
| 14 | ICP.mp. [mp=ab, ti, ot, bt, hw, id, cc, nm, fx, kf, ox, px, rx, ui, sy] |
| 15 | Cerebral blood flow [velocity.mp](http://velocity.mp/). [mp=ab, ti, ot, bt, hw, id, cc, nm, fx, kf, ox, px, rx, ui, sy] |
| 16 | 1 or 2 or 3 or 4 or 5 or 6 or 7 or 8 or 9 or 10 or 11 or 12 or 13 or 14 or 15 |
| 17 | Propofol.mp. [mp=ab, ti, ot, bt, hw, id, cc, nm, fx, kf, ox, px, rx, ui, sy] |
| 18 | 2,6-Diisopropylphenol.mp. [mp=ab, ti, ot, bt, hw, id, cc, nm, fx, kf, ox, px, rx, ui, sy] |
| 19 | Aquafol.mp. [mp=ab, ti, ot, bt, hw, id, cc, nm, fx, kf, ox, px, rx, ui, sy] |
| 20 | Diprivan.mp. [mp=ab, ti, ot, bt, hw, id, cc, nm, fx, kf, ox, px, rx, ui, sy] |
| 21 | Disoprivan.mp. [mp=ab, ti, ot, bt, hw, id, cc, nm, fx, kf, ox, px, rx, ui, sy] |
| 22 | Disoprofol.mp. [mp=ab, ti, ot, bt, hw, id, cc, nm, fx, kf, ox, px, rx, ui, sy] |
| 23 | Fresofol.mp. [mp=ab, ti, ot, bt, hw, id, cc, nm, fx, kf, ox, px, rx, ui, sy] |
| 24 | ICI-35868.mp. [mp=ab, ti, ot, bt, hw, id, cc, nm, fx, kf, ox, px, rx, ui, sy] |
| 25 | Ivofol.mp. [mp=ab, ti, ot, bt, hw, id, cc, nm, fx, kf, ox, px, rx, ui, sy] |
| 26 | Recofol.mp. [mp=ab, ti, ot, bt, hw, id, cc, nm, fx, kf, ox, px, rx, ui, sy] |
| 27 | ICI-35,[868.mp](http://868.mp/). [mp=ab, ti, ot, bt, hw, id, cc, nm, fx, kf, ox, px, rx, ui, sy] |
| 28 | 17 or 18 or 19 or 20 or 21 or 22 or 23 or 24 or 25 or 26 or 27 |
| 29 | 16 and 28 |
| 30 | remove duplicates from 29 |
