# Supplementary material for: Cerebrovascular Response to Propofol, Fentanyl, and Midazolam in Moderate/Severe Traumatic Brain Injury: A Scoping Systematic Review of the Human and Animal Literature
Source: Neurotrauma Rep. 2020 Oct 13;1(1):100–12. doi: 10.1089/neur.2020.0040 (PMC7685293; doi:10.1089/neur.2020.0040)
Supplement: Supplemental data [file Supp_Table3.docx]

Appendix C: Treatment and Cerebrovascular Response – Study Details

| **References** | **Medication and Dose** | **Mean Duration of Dose Administration** | **Technique to Measure Cerebrovascular Response** | **CBF/Cerebrovascular Response** |
| --- | --- | --- | --- | --- |
| ***Human Studies*** | | | | |
| Lee et al^28^ | Phenylephrine to induce a 10 to 15mmHg change in MAP  Propofol: 1 mg/kg | 10 mins | MCAv: Transcranial Doppler  CBF: Xenon^133^ clearance technique  MAP, ICP, pCO_2_ and pO_2_: Technique not mentioned  Vasoreactivity was test different for each group but in general the evaluation of patient response in CBF and MCAv was used as a threshold | **CO_2_ reactivity was induced by lowering end tidal CO_2_ by 6-8mmHg**  -ICP drop by 7mmHg (p<0.01)  -SjvO_2_ by 6% (P<0.001)  -MCAv increased by 3.3±1.6%  -MAP was constant  -Trend to improve vasoreactivity  **Pressure reactivity induced by phenylephrine**  **-**ICP increase of 12mmHg (p<0.001)  -MAP increased by 10-15mmHg  **Metabolic reactivity was induced through propofol burst suppression**  -CPP increase by 5% (p<0.01)  -SjvO_2_ increase by 3% (p<0.01)  -MAP was constant  -MCAv in most models decrease by 30%, CBF also demonstrated a decrease but was not significant  -Trend to deteriorate vasoreactivity with 20% of patient having reduced response  **pCO_2_ and pO_2_ levels were controlled through ventilation** |
| Steiner et al^10^ | Norepinephrine to maintain CPP protocol  Propofol: 3-4mg/kg/h | 4 hrs | MCAv: Transcranial Doppler  ICP: Intraparenchymal transducer  CPP: MAP-ICP  AVDO_2_: Blood samples  pCO_2_ and pO_2_: Blood samples  MAP: Radial artery transducer | **Propofol**  -higher doses decreased MCAv by 8%  -little change to CPP or AVDO_2_  -MAP remained relatively constant  -static rate of autoregulation on average decreased from 56±36 to 28±35% but increased in some patients  **Norepinephrine**  -increased CPP  -increased in MCAv by at least 9 cm/s  -no change to ICP  **pCO_2_ and pO_2_ levels were controlled through ventilation** |
| James et al^34^ | Propofol: 25.5 ug/kg/min  Dexmedetomine: 0.54ug/kg/hr | 2-8.5 days | ICP: Intraventricular catheter  PbtO_2_: Oximetry catheters  CBF: Calculated from ICP/pO_2_  Glucose, lactate, pyruvate, glutamate, glycerol, pCO_2_ and pO_2_: Microdialysis samples  CPP and MAP: Technique not mentioned | **Propofol**  -slight decrease in ICP and no change in PbtO_2_ though both lacked statistical significance  -CPP increased during and fell after injection by about 6% -Lactate/Pyruvate ratio increased drastically after injection  -CBF had minimal changes, based limited response in ICP and pO_2_  **Dexmedetomine**  -a slight increase in ICP with no change in PbtO_2_ though both lacked statistical significance  -CPP fell slightly by 2%  -Lactate/Pyruvate ratio increased drastically after injection  -CBF had minimal changes, based limited response in ICP and pO_2_  **pCO_2_ and pO_2_ levels were controlled through ventilation**  **From ICP and CPP, MAP can be assumed to be near constant** |
| Johnston et al^23^ | Propofol: 3-4mg/kg/h | 4 hrs | CBF: CMRO_2_/AVDO_2_ assuming CMRO_2_ is constant  ICP: Intracranial transducer  pCO_2_ and pO_2_: Microdialysis  CPP and MAP: Technique not mentioned | **Propofol**  -AVDO_2_, ICP and pCO_2_ all slightly decreased as compared to baseline values  -pO_2_ and PbtO_2_ slightly increased  -CPP and Lactate/Pyruvate ratio had little variation  -All changes were not significant  **Effects of propofol on cerebral oxygenation and metabolism after head injury**  **From ICP and CPP, MAP can be assumed to be near constant** |
| Pinaud et al^27^ | Propofol: 2mg/kg then 150ug/kg/min  (3-5ug/ml) | 41.4 ±7.3 mins | rCBF: Xenon^133^ diffusion technique  ICP: Intraventricular catheter  MAP: Radial artery cannula  CPP: MAP-ICP  CVR: CPP/rCBF  AVDO_2,_ pCO_2_ and pO2: Blood samples | **Propofol**  -rCBF decrease by 25%(p<0.01)  -ICP decreased by 18% (p<0.001), this decrease was then inverted after propofol infusion ceased  -CPP dropped by 28% (p<0.001)  -AVDO_2_ decreased by (6%) but was not significant  -CVR increased then decrease as a result from propofol though this was not significant apart from 1 patient  -pCO_2_ remained constant at 33±2 mmHg |
| Tanguy et al^33^ | Propofol: 1mg/kg/h and increased by same increment with 5mg/kg/h being max  Midazolam: 0.03mg/kg/h and increased by 0.01mg/kg/h | 72 hours | ICP: Codman transducer  CBF: Calculated from ICP/pO_2_  pCO_2_ and pO_2_: Microdialysis blood samples  MAP: Arterial transducer | **Propofol**  -ICP of 19±12 mmHg  -pO_2_ of 97±2%  -pCO_2_ of 38±7 mmHg  -CPP of 73±11mmHg  -MAP of 91±11mmHg  -CBF had minimal changes, based limited response in CPP and pO_2_   **Midazolam**  **-**ICP of 20±12 mmHg  -pO_2_ of 98±1 %  -pCO_2_ of 35±10 mmHg  -CPP of 73±11mmHg  -MAP of 100±16mmHg  -CBF had minimal changes, based limited response in CPP and pO_2_  **pCO_2_ and pO_2_ levels were controlled through ventilation**  **No difference was seen in the Lactate/Pyruvate ratio was seen** |
| Albanese et al^24^ | Sufentanil: 1ug/kg then 0.005 ug/kg/min  Alfentanil: 100ug/kg then 0.7ug/kg/min  Fentanyl: 10ug/kg then 0.075 ug/kg/min | 6 mins then 1hr | ICP: Intracranial transducer  CPP: MAP-ICP  AVDO_2_: Blood samples  CBF: Calculated from ICP/pO_2_ and CMRO_2_/AVDO_2_ assuming CMRO_2_ is constant  SvjO_2_: Catheter  pCO_2_ and pO_2_: Blood samples  MAP: Arterial transducer | **Sufentanil, alfentanil and fentanyl**  -initial increase ICP (25%) then after 60 mins ICP returned to baselines  -CPP decreased by 41% (p<0.05)  -SvjO_2_ remained relatively unchanged  -Based on CPP/ SvjO_2,_ CBF was indicated to increase  -Based on CMRO_2_/AVDO_2,_ CBF slight decreased  **pCO_2_ levels were maintained between 32 and 35 torr and CPP stayed between 27 to 37 mmHg**  **pCO_2_ and pO_2_ levels were controlled through ventilation**  **No changes in lactate-oxygen index or MAP** |
| de Nadal et al^26^ | Morphine: 0.2mg/kg  Fentanyl: 2ug/kg | 1mins | CBFv: Transcranial Doppler sonography  AVDO_2_: Blood samples  CBF: CMRO_2_/AVDO_2_ assuming CMRO_2_ is constant  ICP: Intraparenchymal transducer  pCO_2_ and pO_2_: Blood samples  MAP: Arterial transducer  Autoregulation was measured through response of CBF to CO_2_ reactivity | **Morphine**  -slight increase in CBF (10%) with no change in MCAv  -When comparing autoregulation there was little difference in MCAv, however for CBF, impaired autoregulation demonstrated a less overall response (7%) then intact autoregulation (13%)  **Fentanyl**  -slight increase in CBF (10%) and a slight decrease to CBFv (10%)  -There was little difference in impaired vs intact autoregulation in CBF and CBFv response  **All changing in AVDO_2_ were adjusted for pCO_2_ levels**  **MAP remained relatively constant in all groups**  **Slight decrease in CPP associated with ICP increase then constant** |
| de Nadal et al^25^ | Fentanyl: 2 ug/kg | 1 mins | AVDO_2_: Blood samples   End tidal CO_2_: Capnography  ICP: Intraparenchymal transducer  CBF: CMRO_2_/AVDO_2_ assuming CMRO_2_ is constant  pCO_2_ and pO_2_: Blood samples  MAP: Arterial transducer  Autoregulation was measured through response of CBF to CO_2_ reactivity | **Fentanyl**  -ICP: Increased then slowly decrease in both the group with intact and impaired autoregulation -CPP: Moderately decreased by 6% -AVDO_2_ initially decreased (11%) that returned to the baseline at 60mins but was not significant  **MAP showed a similar decrease as CPP**  **All changing in AVDO_2_ were adjusted for pCO_2_ levels** |
| Papazian et al^32^ | Midazolam: 0.15 mg/kg | 30 mins | ICP: Intraparanchymal pressure transducer  CBF: Calculated from ICP/pO_2_  MAP, pCO_2_ and pO_2_: Cannulated radial artery with blood samples | **Midazolam**  -reduced CPP by 26% (p<0.0001)  -non-significant change to ICP>18mmHg before TBI, when ICP<18 mmHg before TBI an increase in ICP was observed (20%)  -CBF had little change apart from mentioned ICP<18mmHg in which case midazolam cause a slight increase in CBF (10%)  **pCO_2_ and pO_2_ were measured and maintained** |
| ***Animal Studies*** | | | | |
| Feuerstein et al^29^ | Isoflurane at 2%  Propofol: 33 to 53 mg/kg/h | 20 mins | rCBF: Laser speckle images  Vessel diameter: Craniotomy and direct visualized vessel change  MAP, pCO_2_ and pO_2_: Cannulated radial artery with blood samples | **Isoflurane**  -rCBF increased initially with injection then returned to baseline after 1 minute (19.8±27.2%)  **Propofol**  -rCBF increased initially with injection then returned to baseline after 1 minute (27.5±38.2%)  -atrial diameter decrease of 50% where isoflurane had no response  **Blood gasses were maintained through ventilation**  **MAP had little change in each group** |
| Kahveci et al^31^ | Propofol: 12mg/kg/hr  Isoflurane: 0.9±0.04% | 180mins | CBFv: Transcranial Doppler flowmetry  ICP: Fiberoptic transducer  MAP, pCO_2_ and pO_2_: Cannulated radial artery with blood samples | **Propofol**  -decrease ICP from 50% (p<0.01)  -CPP decreased by 10%  -no significant change to pO_2_, CBFv or MAP  **Isoflurane**  -no significant effect on CBFv, ICP or pO_2_  -MAP and CPP decrease over time by 30%  **Blood gasses were maintained through ventilation** |
| Bedell et al^30^ | Isoflurane: 1-1.5%  Fentanyl: 50 ug/kg/h | 135mins | CBF: Radiolabel microspheres  ICP: Pressure transducer  CVR: MAP/CBF  pCO_2_ and pO_2_: Blood samples  MAP: Femoral and brachial arteries transducer | **Isoflurane**  -ICP increased  -CPP decreased by 7% then returned to baseline  -MAP, CBF and CVR remain relatively constant  **Fentanyl**  -ICP decreased then slightly increased  -CPP decrease by 30%  -MAP decreased from 30%  -CBF decrease from by 22% at 75 mins then CBF increased to baseline  -CVR decrease by 28%  **EEG, ICP, pCO_2_, pO_2_, pH, and temperature were similar between groups** |
| Statler et al^36^ | Isoflurane at 4% then reduced to 1%  Fentanyl: 50ug/ml then 50ug//kg/h | 4 hrs | ICP: Intraparenchymal transducer  CBF: Not mentioned  pCO_2_ and pO_2_: Blood samples  MAP: Angiocatheter | Fentanyl MAP was higher than isoflurane, however during infusion the MAP and CPP remained constant throughout experiment.  CPP after 4 hrs was greater in fentanyl then then isoflurane group by 10%, but both constant  CBF was 2 to 3 times higher in isoflurane then fentanyl  **pO_2_ and pCO_2_ were controlled by ventilation** |
| AVDO_2_, arterial jugular venous oxygen differences; CBF, cerebral blood flow; CBFv, cerebral blood flow velocity; CMRO_2_, cerebral metabolic rate of oxygen; CO_2_, carbon dioxide; CPP, cerebral perfusion pressure; CVR, cerebrovascular resistance; EEG, electroencephalogram; hrs, hours; ICP, intracranial pressure; min, minutes; MAP, mean arterial pressure; MCAv, middle cerebral artery velocity; mins, minutes; mmHg, millimeters of mercury; PbtO_2_, brain tissue oxygen tension; pCO_2_, partial pressure of carbon dioxide; pO_2_, partial pressure of oxygen; rCBF, regional cerebral blood flow; sec, seconds; SvjO_2_, jugular venous oxygen saturation; TBI, traumatic brain injury; | | | | |
